# iTRAQ-Coupled 2-D LC-MS/MS Analysis of Membrane Protein Profile in *Escherichia coli* Incubated with Apidaecin IB

**DOI:** 10.1371/journal.pone.0020442

**Published:** 2011-06-01

**Authors:** Yusi Zhou, Wei Ning Chen

**Affiliations:** School of Chemical and Biomedical Engineering, College of Engineering, Nanyang Technological University, Singapore, Singapore; University of Osnabrueck, Germany

## Abstract

Apidaecins are a series of proline-rich, 18- to 20-residue antimicrobial peptides produced by insects. They are predominantly active against the Gram-negative bacteria. Previous studies mainly focused on the identification of their internal macromolecular targets, few addressed on the action of apidaecins on the molecules, especially proteins, of bacterial cell membrane. In this study, iTRAQ-coupled 2-D LC-MS/MS technique was utilized to identify altered membrane proteins of *Escherichia coli* cells incubated with one isoform of apidaecins—apidaecin IB. Cell division protease ftsH, an essential regulator in maintenance of membrane lipid homeostasis, was found to be overproduced in cells incubated with apidaecin IB. Its over-expression intensified the degradation of cytoplasmic protein UDP-3-O-acyl-N- acetylglucosamine deacetylase, which catalyzes the first committed step in the biosynthesis of the lipid A moiety of LPS, and thus leaded to the further unbalanced biosynthesis of LPS and phospholipids. Our findings suggested a new antibacterial mechanism of apidaecins and perhaps, by extension, for other proline-rich antimicrobial peptides.

## Introduction

Antimicrobial peptides (AMPs) are a group of relative short (less than 100 amino acids), positive charged peptides produced by a wide variety of organisms as part of their first line of defense [1]. These peptides possess broad-spectrum antimicrobial activity against Gram-positive and Gram-negative bacteria [2], [3], [4], fungi [2], [5], [6], protozoa [2], [7], [8] and viruses [9], [10], [11]. Despite their highly diverse sequences and structural motifs, most of them show a tendency to assume amphiphilic conformation in membrane environments. This trait correlated with their ability to disrupt the bacterial membranes, ultimately leading to lysis of the cells [12]. Besides this membrane disruptive mechanism, a minority of AMPs are bactericidal through a mechanism that is completely devoid of any apparent membrane destabilization [13]. Apidaecins, a series of proline-rich, 18- to 20-residue peptides produced by insects, are one group of such non-membrane-disruptive AMPs [14]. They are predominantly active against Gram-negative bacteria including a wide range of plant-associated bacteria and some human pathogens [15]. They can translocate across cell membrane, penetrate into the cytoplasm, and target essential cellular processes to mediate cell death [14]. As a result, previous studies mainly focused on the identification of their internal macromolecular targets [16], [17], few addressed on the action of apidaecins on the molecules, especially proteins, of bacterial cell membrane, which could attribute to their antibacterial mechanism.

In this study, iTRAQ-coupled 2-D LC-MS/MS technique was utilized to identify altered membrane proteins of *E. coli* incubated with one isoform of apidaecins—apidaecin IB. Cell division protease ftsH (FtsH), an essential regulator in maintenance of membrane lipid homeostasis [18], was found to be overproduced in cells incubated with apidaecin IB for both 1 h and 2 h. Its over-expression intensified the degradation of cytoplasmic protein UDP-3-O-acyl-N-acetylglucosamine deacetylase, which catalyzes the first committed step in the biosynthesis of the lipid A moiety of LPS, and thus leaded to the further unbalanced biosynthesis of LPS and phospholipids. These findings provide new insight into the antibacterial mechanism of apidaecins and perhaps, by extension, for other proline-rich AMPs.

## Results and Discussion

**Figure 1:**
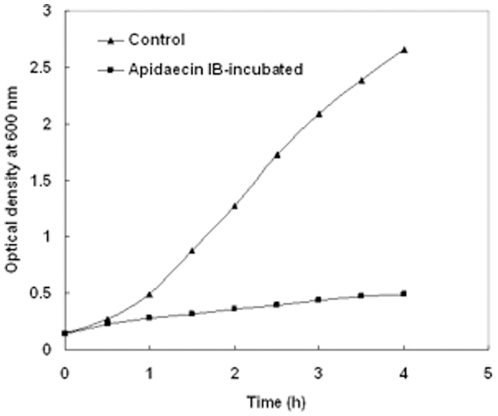
Growth kinetics of *E. coli* incubated with apidaecin IB. Each value represents the mean optical density (OD) readings from two cultures.

The antibacterial activity of apidaecin IB towards *E. coli* cells was evaluated by the broth micro-dilution assay. The MIC of apidaecin IB was found to be as 16 µg/ml. The growth kinetics of cells was subsequently assayed in the presence of ^1^/_10_ MIC of apidaecin IB. Compared to no apidaecin IB control, apidaecin IB started to inhibit *E. coli* growth at 0.5 h after its incubation (Figure 1). Two time points (1 h and 2 h) were therefore chosen in this study.

To investigate how bacterial membrane protein changed in response to apidaecin IB, IM and OM proteins were extracted and identified by iTRAQ-coupled 2D LC-MS/MS analysis, respectively. Thirty-eight IM proteins, 28 OM proteins, and 9 M proteins (M means whether the protein is found in or associated with the inner or outer cell membrane is unknown), were identified in cells incubated with apidaecin IB for both 1 h and 2 h. The Unused ProtScore of those proteins was more than 2 which corresponds to 99% confidence. Among them, 5 IM proteins, 1 OM protein and 2 M proteins showed differential changes with the trends of the changes in 2 h-apidaecin IB-incubated cells were in accordance with those in 1 h-apidaecin IB-incubated cells (Table 1).

**Table 1:** Altered membrane proteins of *E. coli* incubated with apidaecin IB.

AccessionNumber	Protein Name	Unused ProtScore^a^	%Cov^b^	Avg A∶C (±S.D.)^c^1 h	Avg A∶C (±S.D.)2 h	SubcellularLocation^d^	Function
P0AAI3|FTSH	Cell division protease ftsH	18.03	53.26	1.67±0.16	2.23±0.37	IM	Metalloproptease
P31224|ACRB	Acriflavine resistance protein B	6.54	29.17	0.87±0.02	0.77±0.01	IM	Drug efflux
P09127|HEMX	Putative uroporphyrinogen-III C-methyltransferase	6	27.23	1.14±0.14	1.44±0.01	IM	Porphyrin biosynthesis
P20966|PTFBC	PTS system fructose-specific EIIBC component	5.8	28.6	0.87±0.04	0.64±0.00	IM	Fructose transport
P15877|DHG	Quinoprotein glucose dehydrogenase	3.56	27.39	1.45±0.01	1.53±0.09	IM	Energy conservation
P0A935|MLTA	Membrane-bound lytic murein transglycosylase A	2.19	23.84	1.12±0.01	1.17±0.07	OM	Murein degradation
P0ADA5|YAJG	Uncharacterized lipoprotein yajG	9.68	53.13	1.35±0.08	1.66±0.25	M	Unknown
P11557|DAMX	Protein damX	6	25.23	1.00±0.01	1.27±0.06	M	Interferes with cell division

a Unused ProtScore is a measure of the protein confidence for a detected protein, calculated from the peptide confidence for peptides from spectra that are not already completely “used” by higher scoring winning proteins.

ProtScore = −log (1-Percent Confidence/100).

The corresponding Percent Confidence of ProScore 2.0 is 99%.

b The percentage of matching amino acids from identified peptides having confidence greater than 0, divided by the total number of amino acids in the sequence.

c The ratio of protein production level in apidaecin-incubated cells to control cells.

d Subcellular location: IM, inner membrane; OM, Outer membrane; M, membrane with whether the protein is found in or associated with the inner or outer cell membrane is not known.

**Figure 2:**
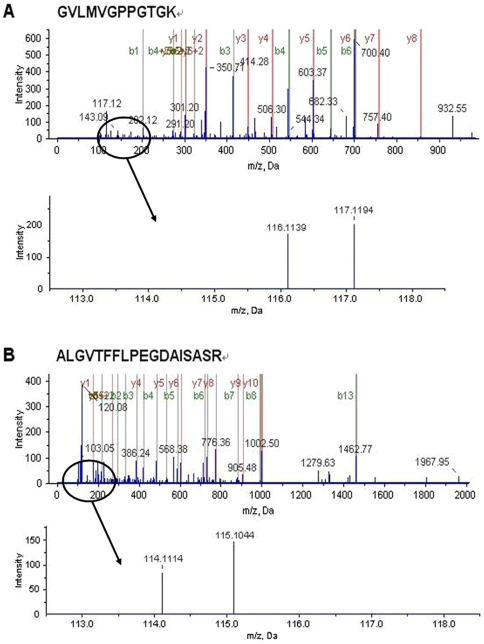
Representative MS/MS spectra of FtsH. (A) A peptide, GVLMVGPPGTGK, derived from FtsH in cells incubated without and with apidaecin IB for 1 h. (B) a peptide, ALGVTFFLPEGDAISASR, derived from FtsH in cells incubated without and with apidaecin IB for 2 h. The ion assignments are as follows: iTRAQ tags 116, Control 1 h; iTRAQ tags 117, Apidaecin IB-incubated 1 h; iTRAQ tags 114, Control 2 h; iTRAQ tags 115, Apidaecin IB-incubated 2 h.

**Figure 3:**
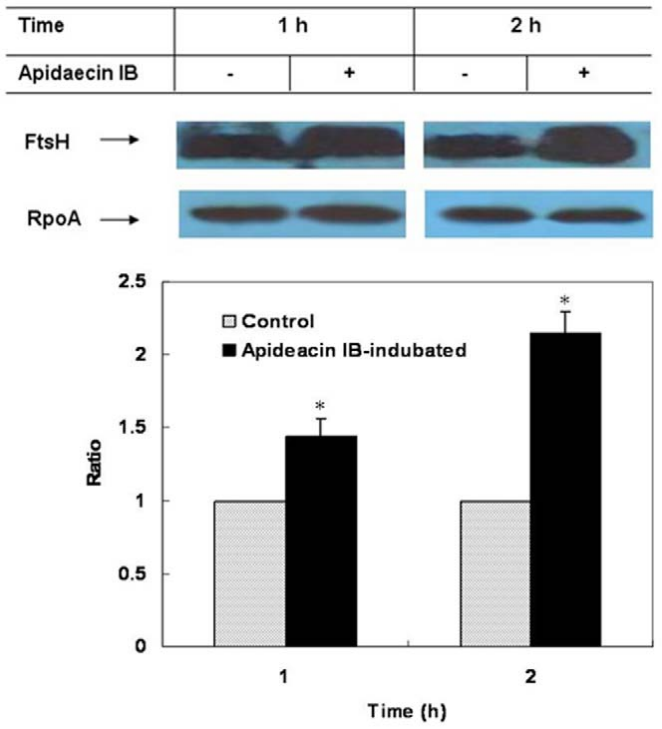
Western blot analysis of FtsH in *E. coli* incubated with apidaecin IB. The relative densitometric intensity of FtsH to RpoA in cells without incubation of apidaecin IB was adjusted to 1 and that in cells incubated with apidaecin IB was normalized accordingly. Asterisk indicates *p*<0.05.

One of the altered membrane proteins, cell division protease ftsH, captured our attention. FtsH was overproduced in both 1 h and 2 h-apidaecin-incubated cells, with the increase in the latter was greater than that in the former (Table 1). The representative MS/MS spectra of peptides derived from FtsH are shown in Figure 2. The changes of FtsH were further validated by western blot analysis (Figure 3).

**Figure 4:**
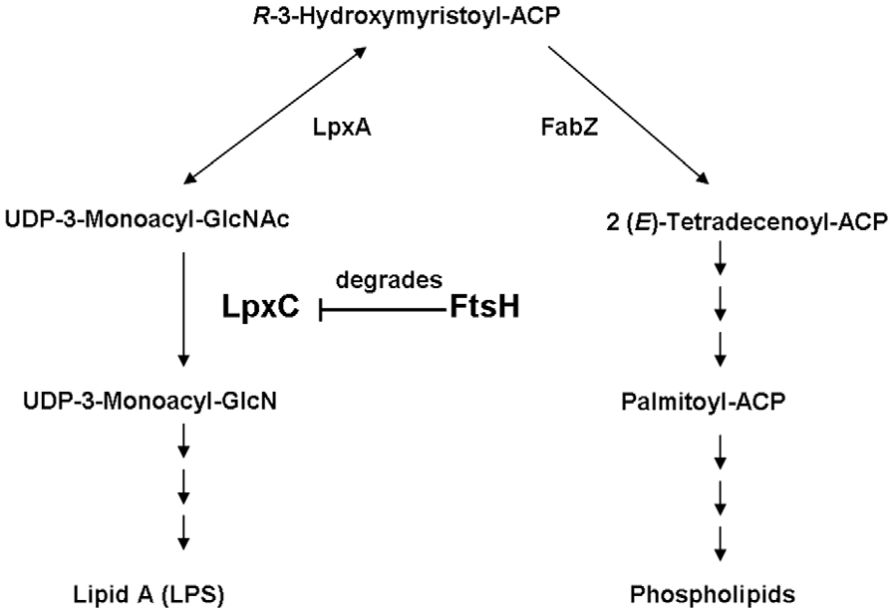
Schematic representation of biosynthetic pathways of membrane lipid components. Functions of FtsH in the regulation of biosynthesis of LPS and phospholipids are drawn. ACP, acyl carrier protein; GlcNAc, *N*-acetylglucosamine; GlcN, glucosamine.

**Figure 5:**
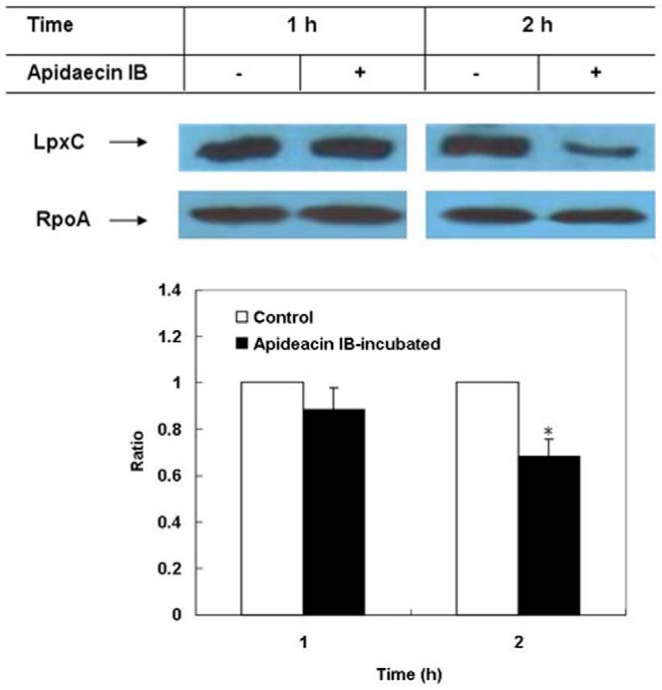
Western blot analysis of LpxC in *E. coli* incubated with apidaecin IB. The relative densitometric intensity of LpxC to RpoA in cells without incubation of apidaecin IB was adjusted to 1 and that in cells incubated with apidaecin IB was normalized accordingly. Asterisk indicates *p*<0.05.

**Figure 6:**
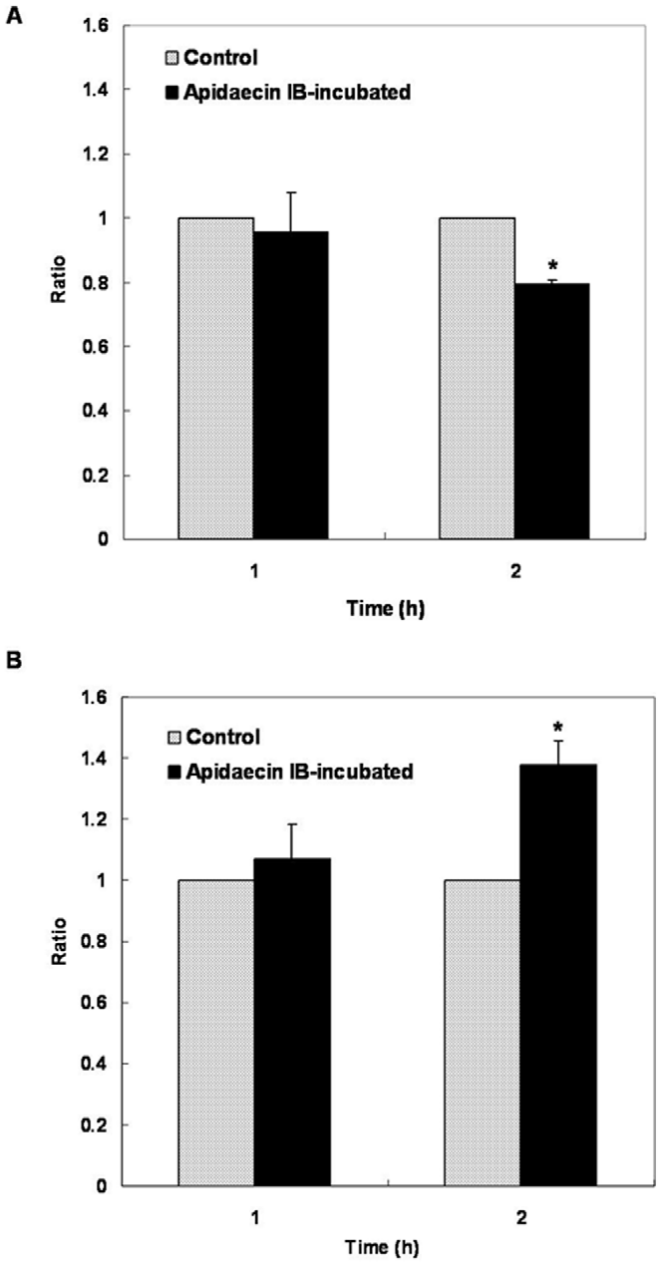
Analysis of LPS (A) and phospholipids (B) in *E. coli* incubated with apidaecin IB. LPS and phospholipids were determined by measuring KDO and phosphate, respectively. Values for cells without incubation of apidaecin IB were adjusted to 1 and those for cells incubated with apidaecin IB were normalized accordingly. Asterisk indicates *p*<0.05.

Gram-negative bacteria have two membranes—IM and OM. The IM is a phospholipid bilayer, and the OM is an asymmetrical bilayer consisting of phospholipids and LPS in the inner and outer leaflet, respectively. The synthesis of LPS and phospholipids must be properly balanced, which is critical for cell viability. The same reaction precursor (*R*-3-hydroxymyristoyl-ACP) is used by LpxC for the biosynthesis of the lipid A moiety of LPS (LpxC catalyzes the first committed step) and by FabZ ((3*R*)-hydroxymyristoyl-[acyl-carrier-protein] dehydratase) for the synthesis of fatty acid (Figure 4) [19], [20], [21], [22], [23], [24]. Thus the balance of these enzymes is important to maintain a proper LPS/phospholipids ratio. FtsH is the sole, ATP-dependent, growth-essential protease of *E. coli* [25]. Its essentiality lies in its function in keeping a proper LpxC/FabZ ratio by degrading LpxC [18], [26]. The overproduction of FtsH in this study would probably lead to the changes in the cellular level of LpxC. We therefore analyzed LpxC by Western blotting. The results showed that no significant changes in the amount of LpxC in cells incubated with apidaecin IB for 1 h; however, the amount of LpxC markedly decreased in cells incubated with apidaecin IB for 2 h (Figure 5). We then did LPS and phospholipids analysis. The results showed that the amount of LPS markedly decreased in cells incubated with apidaecin IB for 2 h, in contrast, the amount of phospholipid significantly increased (Figure 6).

**Figure 7:**
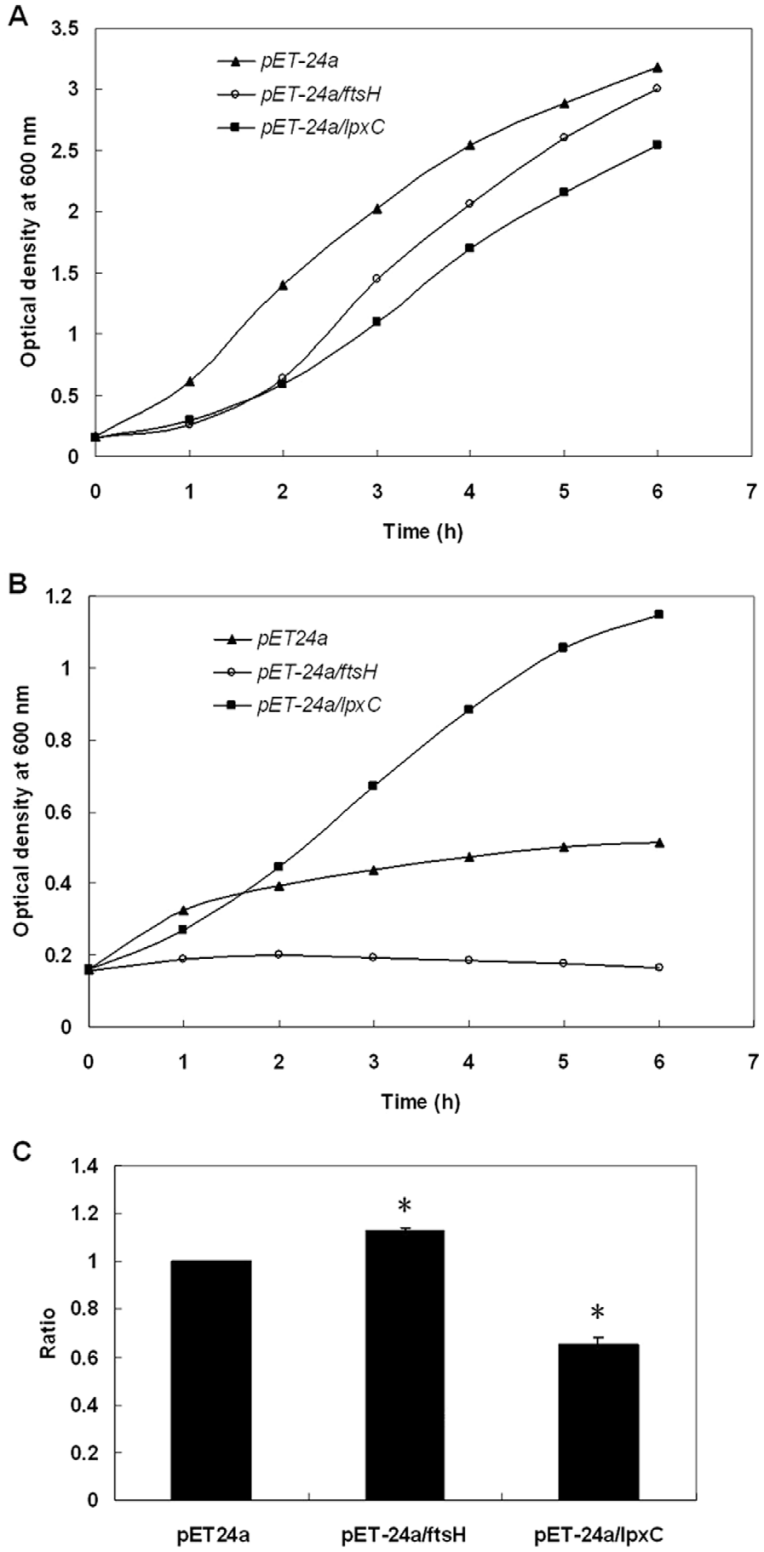
Effect of FtsH and LpxC overexpression on the growth of *E. coli* incubated with apidaecin IB. (A) Growth curve of *E. coli* separately harboring pET-24a, pET-24a/*ftsH* and pET-24a*/lpxC* plasmids without apidaecin IB incubation. Each value represents the mean OD readings from two cultures. (B) Growth curve of *E. coli* separately harboring pET-24a, pET-24a/*ftsH* and pET-24a*/lpxC* plasmids with apidaecin IB incubation. Each value represents the mean OD readings from two cultures. (C) Inhibition rate from the 6 h cultures. Inhibition rate was calculated from bacterial OD. Inhibition rate of *E. coli* harboring pET-24a plasmid was adjusted to 1 and those of cells harboring pET-24a/ftsH and pET-24a/lpxC plasmids were normalized accordingly. Asterisk indicates p<0.05.

**Figure 8:**
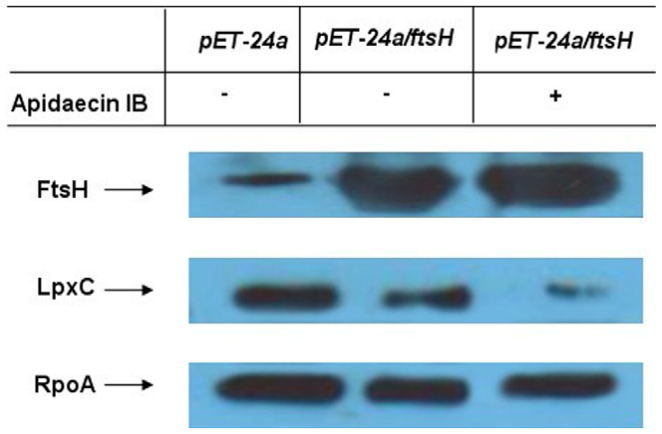
Effect of FtsH overexpression on the production of LpxC in *E.coli* incubated with apidaecin IB.

Moreover, we investigated the characterization of FtsH and LpxC in response to apidaecin IB incubation by using gene-overexpression strains. *ftsH* and *lpxC* were separately cloned into a pET-24a vector and expressed in *E. coli* BL21 (DE3) cells. Cells containing the pET-24a, pET-24a/*ftsH* and pET-24a*/lpxC* plasmid were then separately cultured in LB-kanamycin medium. After IPTG induction for 3 h, cell suspensions were diluted to obtain a concentration of 5×10^5^ CFUs/ml and then incubated without and with ^1^/_10_ MIC of apidaecin IB. Cell growth was checked by measuring OD_600_ in interval of 1 h (Figure 7 A, B). The inhibition rate of these bacteria separately harboring pET-24a, pET-24a/*ftsH* and pET-24a*/lpxC* was obtained by comparing the OD_600_ of the 6 h cultures. The results showed that overexpression of FtsH enhanced the inhibition effect of apidaecin IB on cells; in contrast, overexpression of LpxC can significantly alleviate this effect (Figure 7 C). Cellular proteins of the 6 h cultures were also isolated; the LpxC level in ftsH overexpression cells was further analyzed by western blotting. The results indicated that only with the incubation of apidaecin IB, the overexpression of ftsH cause the decrease in the cellular level of LpxC (Figure 8). Collectively, the data suggest that apidaecin IB act against *E. coli* by overexpressing FtsH to intensify the degradation of LpxC. As *R*-3-hydroxymyristoyl-ACP is used by both LpxC for the synthesis of LPS and by FabZ for the synthesis of phospholipids, the over-degradation of LpxC will leave more *R*-3-hydroxymyristoyl-ACP to FabZ, and ultimately lead to the unbalance LPS/phospholipids ratio and the breaking of membrane lipid homeostasis (Figure 4).

Apidaecins are the largest group of proline-rich AMPs known to date [13]. The apidaecins and other short proline-rich AMPs attract particular interest because of their special antibacterial mechanism that is non-membrane-disruptive [14]. They can translocate across cell membrane, penetrate into the cytoplasm, and target essential cellular processes to mediate cell death [14]. Previous studies on the antibacterial mechanism of apidaecins identified that apidaecins kill the bacteria by inhibiting heat shock protein DnaK's two major functions: the ATPase activity and refolding of misfolded proteins [17]. However, it is possible that these peptides inactivate bacteria by other mechanisms. Overproduction of FtsH and the resulting intensified degradation of LpxC in response to apidaecin incubation found in this study could be involved in a new antibacterial mechanism of apidaecins. However, further studies are required to indentify the reason of FtsH overproduction and other specific targets of FtsH except LpxC to fully understand this new mechanism.

In conclusion,iTRAQ-coupled 2-D LC-MS/MS technique was utilized to identify altered membrane proteins of *E. coli* incubated with apidaecin IB. FtsH was identified to be overproduced in apidaecin IB-incubated cell. The overproduction of FtsH resulted in the intensified degradation of LpxC and the following unbalanced LPS/phospholipids ratio. Our findings shed new lights on the antibacterial mechanism analysis of apidaecins and perhaps, by extension, for other proline-rich AMPs.

## Materials and Methods

### Bacterial strain and culture

The bacterial strain used in this work was *Escherichia coli* ATCC25922 obtained from the American Type Culture Collection (Rockville, MD). Frozen *E. coli* stock was streaked on to Mueller-Hinton (MH) agar plates and grown at 37°C. Cells from a single colony were inoculated into MH broth and cultured overnight at 37°C with shaking at 225 rpm for subsequent experiments.

### Minimal inhibitory concentration (MIC) assay

The MIC of apidaecin IB was determined as described previously [27]. An aliquot of fresh overnight culture was inoculated into MH broth and incubated at 37°C with shaking at 225 rpm until the optical density at 600nm (OD_600_) of the undiluted culture was between 0.2 and 0.4. Cell suspension was diluted to obtain a concentration of 5×10^5^ colony-forming units (CFUs)/ml. Apideacin IB (AnaSpec Corp., USA) was diluted in 0.01% acetic acid buffer to obtain a concentration of 1280 µg/ml. The diluted cell suspension (100 µl) and the serial two-fold dilution of the peptide solution (11 µl) were distributed in each well of round-bottomed, 96-well microtiter plate. Growth of cells in the plate was determined by visual inspection after 16–20 h incubation at 37°C. The MIC was defined as the lowest concentration that inhibited visible growth of the tested isolate.

### Growth kinetics of *E. coli* incubated with apidaecin IB

An aliquot of fresh overnight culture was inoculated into MH broth and incubated at 37°C with shaking at 225 rpm until OD_600_ of the undiluted culture was between 0.2 and 0.4. Cell suspension was diluted to obtain a concentration of 5×10^5^ CFUs/ml and then incubated without and with ^1^/_10_ MIC of apidaecin IB. Cell growth was checked by measuring OD_600_ at 0, 0.5, 1, 1.5, 2, 2.5, 3, 3.5 and 4 h.

### Cytoplasmic and membrane proteins isolation

*E. coli* cells (5×10^5^ CFUs/ml) were incubated with ^1^/_10_ MIC of apidaecin IB for 1 and 2 h. The cytoplasmic and membrane proteins were isolated as described previously with a slightly modification [28]. Briefly, the cells were harvested by centrifugation at 3000×g for 10 min at 4°C and lysed in lysis buffer (50 mM NaCl, 5 mM DTT, 1 mM PMSF and 50 mM Tris·Cl, pH 8.0) by intermittent sonication. Unbroken cells were removed by centrifugation at 3000×g for 10 min at 4°C. The supernatants containing the cytoplasmic proteins were collected by centrifugation at 120,000×g for 60 min at 4°C. The resulting pellets were resuspended in inner membrane solubilization buffer (1% Sarkosyl, 100 mM NaCl and 50 mM Tris·Cl, pH 8.0) and incubated at room temperature for 60 min with gentle shaking. The supernatants containing the solubilized inner membrane proteins were collected by centrifugation at 120,000×g for 60 min at 4°C again. The pellets were resuspended in Milli-Q water and centrifuged for up to three times. The resulting pellets were resuspended in outer membrane solubilization buffer (3% n-octylpolyoxyethylene, 150 mM NaCl, 50 mM EDTA, 10 mM DTT, 0.1 mg/ml hen egg lysozyme and 50 mM Tris·Cl, pH 8.0) and incubated at room temperature for 60 min with stirring. The supernatants containing the solubilized outer membrane proteins were collected by centrifugation as above. The concentration of cytoplasmic, inner and outer membrane proteins were determined by Bradford assay. Standard curves were made using γ-globulin as a control.

### iTRAQ Labeling

Proteins from each sample (100 µg) were precipitated by the addition of four volumes of cold acetone at −20°C for 2 h. The precipitated pellets were reduced, cysteine blocked, digested and labeled with respective isobaric tags using iTRAQ reagent Multiplex kit (Applied Biosystems Inc., CA, USA) according to manufacturer's protocol. The sample labeling was as follows: iTRAQ tags 114, Control 2 h; iTRAQ tags 115, Apidaecin IB-incubated 2 h; iTRAQ tags 116, Control 1 h; iTRAQ tags 117, Apidaecin IB-incubated 2 h. The samples were then pooled for LC-MS/MS system.

### LC-MS/MS analysis

iTRAQ-labeled peptide mixtures were analyzed by 2-dimentional nanoflow LC system (Agilent Technologies Inc., USA) interfaced with QSTAR XL mass spectrometer (Applied Biosystems Inc., USA) as described previously [29], [30], [31], [32], [33]. The peptide mixture was loaded into a PolySulfoethyl A strong cation exchange (SCX) column (50×0.32 mm, 5 µm, PolyLC Inc., USA) and fractioned by ten salt steps with 5 µl of buffers (10, 20, 30, 40, 50, 60, 80, 100, 300, and 500 mM KCl) in the first dimension. The peptides eluted from the SCX column were concentrated and desalted in a ZORBAX 300SB C18 reversed-phase (RP) column (5×0.3 mm, 5 µm, Agilent Technologies Inc., USA). The second dimensional chromatographic separation was carried out with a ZORBAX 300SB C18 RP column (50×0.075 mm, 3.5 µm, Agilent Technologies Inc., USA) directly into a PicoFrit nanospray tip (New Objective, USA) operating at a flow rate of 500 nl/min with a 100-min gradient. The mass spectrometer was operated at a nanospray voltage of 2.2 kV. Data were acquired in the positive ion mode with a selected mass range of 300–2000 m/z. Up to two peptides with +2 to +4 charged were selected for MS/MS using dynamic exclusion. The automatic rolling collision energy was used to promote fragmentation. The peak areas of the iTRAQ reporter ions reflect the relative abundance of the proteins in the sample.

### Mass Spectrometric Data Analysis

The identification and quantification of the proteins were performed using ProteinPilot Software 3.0 (Applied Biosystems Inc., USA). The Paragon algorithm in the ProteinPilot software was used for the peptide identification and further processed by Pro Group algorithm where isoform-specific quantification was adopted to trace the differences between expressions of various isoforms. The defined parameters were as follows: (i) Sample Type, iTRAQ 4-plex (Peptide Labeled); (ii) Cysteine alkylation, MMTS; (iii) Digestion, Trypsin; (iv) Instrument, QSTAR ESI; (v) Special factors, None; (vi) Species, *Escherichia coli*; (vii) Specify Processing, Quantitate, Bias correction (viii) ID Focus, Biological modifications; (ix) Database, UniProt_sprot_20070123; (x) Search effort, thorough. The peptide for quantification was automatically selected by Pro Group algorithm, with the criterion that the peptide was usable for quantitation, identified with good confidence, and not shared with another protein identified with higher confidence, to calculate the reporter peak area, error factor (EF) and *p*-value. The resulting proteins met the criteria that Unused ProtScore (a measure of the protein confidence calculated as ProtScore = −log (1-Percent Confidence/100)) was greater than 2.0 (the corresponding Percent Confidence is 99%), the fold change was greater than 1.1 or less than 0.9, and the EF and *p*-value of the fold change were less than 2 and 0.05 respectively were considered for further analysis.

### Western blot analysis

Rabbit antisera to FtsH and LpxC were produced by Invitrogen Corp., USA. The specificity of anti-FtsH and LpxC was validated. These antisera were used as the primary antibodies. Western blotting was performed as described previously [34]. Briefly, equal amounts of proteins were separated with 8% SDS-PAGE. The proteins were electro-transferred to PVDF membranes (Bio-Rad Laboratories Inc., USA), which were then probed with primary antibodies. Horseradish peroxidase (HRP)-conjugated goat anti-rabbit antibody (Santa Cruz Biotechnology Inc., USA) was used as the secondary antibody. The results were visualized using SuperSignal West Pico Chemiluminescent Substrate (Thermo Fisher Scientific Inc., USA). The expression of DNA-directed RNA polymerase subunit alpha (RpoA) was used as a loading control. The densitometric intensity of protein bands was measured using Quantity One software (Bio-Rad Laboratories Inc., USA).

### LPS and phospholipids analysis

LPS was extracted by using LPS extraction kit (iNtRON Biotechnology, Korea) according to manufacturer's protocol and quantified by measuring 2-keto-3-deoxyheptonic acid (KDO) as described previously [35]. Phospholipids were extraction by a method described previously with a slight modification [36]. In brief, the cell pellets were resuspended in 2 volumes of Milli-Q water and mixed with 7.5 volumes organic solvent mixture (methanol-chloroform 2∶1, v/v). This suspension was incubated at room temperature for 2 h with periodic vortexing. After centrifugation at 3000×g for 10 min, extract from the top was removed and mixed with half volume of chloroform and Milli-Q water. After thorough vortexing, the mixture was centrifuged again. Extract from the lower chloroform phase was removed and quantitated by phosphate assay as described previously [37].

### Gene cloning and overexpression

The gene encoding *E. coli* FtsH and LpxC was amplified by PCR, using the appropriate forward and reverse oligonucleotide primers. The forward primer (5′-CCGGAATTCATGGCGAAAAACCTAATAC-3′ for *ftsH* and 5′- CCGGAATTCATGATCAAACAAAGGACAC-3′ for *lpxC*) introduced an *Eco*RI site and the reverse primer (5′-CCGCTCGAGTTACTTGTCGCCTAACTGC-3′ for *ftsH* and 5′-CCGCTCGAGTTATGCCAGTACAGCTGAAGG-3′ for *lpxC*) introduced an *Xho*I site downstream from the stop codon. The PCR product was digested with the corresponding restriction enzymes, isolated from an agarose gel, and ligated into pET-24a (EMD Chemicals, USA). The ligation mixture was used to transform *E. coli* DH5α-competent cells, and the colonies were selected on agar plates containing kanamycin (50 µg/ml). After verifying the DNA sequence, plasmid DNA was transformed into *E. coli* BL21 (DE3) cells. Expression of the two proteins is induced by the addition of IPTG (1 mM), which provides a tightly regulated bacterial expression system

### Statistical analysis

Unless indicated in the figure legends, all experiment were replicated three times. The statistical significance was assessed by Student's t-tests. A *p*-value<0.05 was considered significant.

## References

[1] Hancock RE, Lehrer R (1998). Cationic peptides: a new source of antibiotics.. Trends Biotechnol.

[2] Zasloff M (1987). Magainins, a class of antimicrobial peptides from Xenopus skin: isolation, characterization of two active forms, and partial cDNA sequence of a precursor.. Proc Natl Acad Sci U S A.

[3] Nakamura T, Furunaka H, Miyata T, Tokunaga F, Muta T (1988). Tachyplesin, a class of antimicrobial peptide from the hemocytes of the horseshoe crab (Tachypleus tridentatus). Isolation and chemical structure.. J Biol Chem.

[4] Selsted ME, Novotny MJ, Morris WL, Tang YQ, Smith W (1992). Indolicidin, a novel bactericidal tridecapeptide amide from neutrophils.. J Biol Chem.

[5] Iwanaga S, Muta T, Shigenaga T, Seki N, Kawano K (1994). Structure-function relationships of tachyplesins and their analogues.. Ciba Found Symp.

[6] Ahmad I, Perkins WR, Lupan DM, Selsted ME, Janoff AS (1995). Liposomal entrapment of the neutrophil-derived peptide indolicidin endows it with in vivo antifungal activity.. Biochim Biophys Acta.

[7] Morvan A, Iwanaga S, Comps M, Bachere E (1997). In Vitro Activity of the Limulus Antimicrobial Peptide Tachyplesin I on Marine Bivalve Pathogens.. J Invertebr Pathol.

[8] Aley SB, Zimmerman M, Hetsko M, Selsted ME, Gillin FD (1994). Killing of Giardia lamblia by cryptdins and cationic neutrophil peptides.. Infect Immun.

[9] Aboudy Y, Mendelson E, Shalit I, Bessalle R, Fridkin M (1994). Activity of two synthetic amphiphilic peptides and magainin-2 against herpes simplex virus types 1 and 2.. Int J Pept Protein Res.

[10] Murakami T, Niwa M, Tokunaga F, Miyata T, Iwanaga S (1991). Direct virus inactivation of tachyplesin I and its isopeptides from horseshoe crab hemocytes.. Chemotherapy.

[11] Robinson WE, McDougall B, Tran D, Selsted ME (1998). Anti-HIV-1 activity of indolicidin, an antimicrobial peptide from neutrophils.. J Leukoc Biol.

[12] Tossi A, Sandri L, Giangaspero A (2000). Amphipathic, alpha-helical antimicrobial peptides.. Biopolymers.

[13] Markossian KA, Zamyatnin AA, Kurganov BI (2004). Antibacterial proline-rich oligopeptides and their target proteins.. Biochemistry (Mosc).

[14] Li WF, Ma GX, Zhou XX (2006). Apidaecin-type peptides: biodiversity, structure-function relationships and mode of action.. Peptides.

[15] Casteels P, Ampe C, Jacobs F, Vaeck M, Tempst P (1989). Apidaecins: antibacterial peptides from honeybees.. EMBO J.

[16] Castle M, Nazarian A, Yi SS, Tempst P (1999). Lethal effects of apidaecin on Escherichia coli involve sequential molecular interactions with diverse targets.. J Biol Chem.

[17] Otvos L, O I, Rogers ME, Consolvo PJ, Condie BA (2000). Interaction between heat shock proteins and antimicrobial peptides.. Biochemistry.

[18] Ogura T, Inoue K, Tatsuta T, Suzaki T, Karata K (1999). Balanced biosynthesis of major membrane components through regulated degradation of the committed enzyme of lipid A biosynthesis by the AAA protease FtsH (HflB) in Escherichia coli.. Mol Microbiol.

[19] Anderson MS, Robertson AD, Macher I, Raetz CRH (1988). Biosynthesis of lipid-A in Escherichia coli-identification of UDP-3-O-[(R)-3- hydroxymyristoyl]-alpha-D-glucosamine as a precursor of UDP-N,2, O-3-bis[(R)-3-hydroxymyristoyl]-alpha-D-glucosamine.. Biochemistry.

[20] Anderson MS, Bull HG, Galloway SM, Kelly TM, Mohan S (1993). UDP-N-Acetylglucosamine acyltransferase of Escherichia coli-the 1^st^ step of endotoxin biosynthesis is thermodynamically unfavorable.. Journal of Biological Chemistry.

[21] Anderson MS, Raetz CRH (1987). Biosynthsis of lipid-A precursors in Escherichia coli-a cytoplasmic acyltransferase that converts UDP-N-Acetylglucosamine to UDP-3-O-(R-3-hydroxymyristoyl)-N-acetylglucosamine.. Journal of Biological Chemistry.

[22] Anderson MS, Bulawa CE, Raetz CRH (1985). The biosynthesisi of gram-negative endotoxin-formation of lipid A precursors from UDP-GlcNAc in extracts of Escherichia coli.. Journal of Biological Chemistry.

[23] Heath RJ, Rock CO (1996). Roles of the FabA and FabZ beta-hydroxyacyl-acyl carrier protein dehydratases in Escherichia coli fatty acid biosynthesis.. Journal of Biological Chemistry.

[24] Mohan S, Kelly TM, Eveland SS, Raetz CRH, Anderson MS (1994). An Escherichia coil gene (FabZ) encoding (3R)-hydroxymyristoyl acyl carrier protein dehydrase-relation to FabA and suppression of mutations in lipid A biosynthesis.. Journal of Biological Chemistry.

[25] Tomoyasu T, Gamer J, Bukau B, Kanemori M, Mori H (1995). Escherichia coi FtsH is a membrane-bound, ATP-dependent protease which degrades the heat-shock transcription factor sigma (32).. Embo Journal.

[26] Fuhrer F, Muller A, Baumann H, Langklotz S, Kutscher B (2007). Sequence and length recognition of the C-terminal turnover element of LpxC, the membrane-bound FtsH a soluble substrate of protease.. Journal of Molecular Biology.

[27] Wiegand I, Hilpert K, Hancock REW (2008). Agar and broth dilution methods to determine the minimal inhibitory concentration (MIC) of antimicrobial substances.. Nature Protocols.

[28] Arnold T, Linke D (2008). The use of detergents to purify membrane proteins.. Curr Protoc Protein Sci Chapter.

[29] Zhou YS, Lamrani M, Chan-Park MB, Leong SS, Chang MW (2010). iTRAQ-coupled two-dimensional liquid chromatography/tandem mass spectrometric analysis of protein profile in Escherichia coli incubated with human neutrophil peptide 1–potential in antimicrobial strategy.. Rapid Commun Mass Spectrom.

[30] Sui J, Tan TL, Zhang J, Ching CB, Chen WN (2007). iTRAQ-coupled 2D LC-MS/MS analysis on protein profile in vascular smooth muscle cells incubated with S- and R-enantiomers of propranolol: possible role of metabolic enzymes involved in cellular anabolism and antioxidant activity.. J Proteome Res.

[31] Sui J, Zhang J, Tan TL, Ching CB, Chen WN (2008). Comparative proteomics analysis of vascular smooth muscle cells incubated with S- and R-enantiomers of atenolol using iTRAQ-coupled two-dimensional LC-MS/MS.. Mol Cell Proteomics.

[32] Zhang J, Sui J, Ching CB, Chen WN (2008). Protein profile in neuroblastoma cells incubated with S- and R-enantiomers of ibuprofen by iTRAQ-coupled 2-D LC-MS/MS analysis: possible action of induced proteins on Alzheimer's disease.. Proteomics.

[33] Zhang J, Niu D, Sui J, Ching CB, Chen WN (2009). Protein profile in hepatitis B virus replicating rat primary hepatocytes and HepG2 cells by iTRAQ-coupled 2-D LC-MS/MS analysis: Insights on liver angiogenesis.. Proteomics.

[34] Niu D, Sui J, Zhang J, Feng H, Chen WN (2009). iTRAQ-coupled 2-D LC-MS/MS analysis of protein profile associated with HBV-modulated DNA methylation.. Proteomics.

[35] Karkhanis YD, Zeltner JY, Jackson JJ, Carlo DJ (1978). A new and improved microassay to determine 2-keto-3-deoxyoctonate in lipopolysaccharide of Gram-negative bacteria.. Anal Biochem.

[36] Kloser A, Laird M, Deng M, Misra R (1998). Modulations in lipid A and phospholipid biosynthesis pathways influence outer membrane protein assembly in Escherichia coli K-12.. Mol Microbiol.

[37] Chen PS, Toribara TY, Warner H (1956). Microdetermination of phosphorus.. Analytical Chemistry.

